# Toll Like Receptor 9 (*TLR9*) Polymorphism G520R in Sheep Is Associated with Seropositivity for *Small Ruminant Lentivirus*

**DOI:** 10.1371/journal.pone.0063901

**Published:** 2013-05-15

**Authors:** Theologia Sarafidou, Costas Stamatis, Georgia Kalozoumi, Vassiliki Spyrou, George C. Fthenakis, Charalambos Billinis, Zissis Mamuris

**Affiliations:** 1 Department of Biochemistry and Biotechnology, University of Thessaly, Larissa, Greece; 2 Department of Animal Production, Technological Educational Institute, Larissa, Greece; 3 Faculty of Veterinary Medicine, University of Thessaly, Karditsa, Greece; Northeast Agricultural University, China

## Abstract

Infectious diseases of sheep are of major economic importance causing direct and indirect losses. Among the major sheep infectious agents are *Small Ruminant Lentivirus*, *Chlamydophila abortus* and *Mycobacterium avium* subsp. *paratuberculosis* infections, mainly due to their worldwide distribution and economic impact that they cause. Based on the differential susceptibility to infectious diseases between and within breeds and on the recent findings regarding the putative involvement of TLR9 in disease susceptibility, the aim of this study was to evaluate the levels of nucleotide variation of TLR9 and its mediator MyD88 in three sheep flocks originated from different breeds and assess their possible association with seropositivity/seronegativity for different infectious agents. The analysis indicated that the change of G to R at codon 520 of TLR9 polypeptide shows a significant association with *Small Ruminant Lentivirus* seropositivity. This amino-acid substitution, which can result in polarity change, might influence structure and function of LRR17, interfering with ligand binding and thus could be used in studies investigating susceptibility/resistance to *Small Ruminant Lentivirus* infections in sheep.

## Introduction

Infectious diseases adversely affect health and production of sheep, with their control measures including administration of antimicrobial drugs, which may lead in development of antibiotic-resistant bacterial strains, vaccination of animals, which must be performed strategically in order to be effective, and management measures, which rely on the farmer’s compliance [1]. Nevertheless, there is evidence of genetic variation between sheep breeds and individuals in their susceptibility to infections. This can offer an opportunity for genetic selection for resistance to infectious diseases, through identification of responsible genes and study of host-resistance mechanisms.

Among the important infective agents of sheep are *Small Ruminant Lentivirus*, *Chlamydophila abortus* and *Mycobacterium avium* subsp. *paratuberculosis*, all with a worldwide distribution. *Small Ruminant Lentivirus* infections, responsible for significant economic losses, due to decreased milk production and early culling age of the animals [2], are caused by five groups of the virus (A, B, C, D, E), which affect a variety of tissues. *C. abortus* is an abortifacient agent, with zoonotic importance, responsible for significant financial consequences [3]. Ovine paratuberculosis, caused by *M. avium* subsp. *paratuberculosis*, results in increased mortality, morbidity and production losses in flocks and has a difficult diagnosis, as infected animals may not develop clinical signs of the disease for several years [4].

Cumulative evidence indicates that Toll-Like Receptors (TLRs), which are key components of pathogen recognition and innate immunity mechanism activation, have been associated with increased susceptibility to various infective agents in humans (e.g., AIDS [5], lepromatous leprosy [6]) or animals (e.g., paratuberculosis in cattle [7], [8], salmonellosis in pigs [9], brucellosis in sheep [10]). TLRs are type I integral membrane glycoproteins (90–115 kD), consisting of an ectodomain with 19–25 tandem copies of a leucine rich repeat (LRR) motif and a cytoplasmic Toll/IL1R (TIR) domain, linked by a transmembrane domain [11]. These receptors bind lipids, lipoproteins, proteins and nucleic acids through their ectodomains, whilst adaptor-protein recruitment takes place via their TIR domain in order to promote signal transduction [11].

Association of TLR molecules with disease susceptibility in sheep has been suggested, based on their differential expression patterns between infected and non-infected animals and the results of association analysis of specific polymorphic sites with disease status; So far, SNPs in TLR1, TLR2, TLR4, TLR6 and TLR10 have produced suggestive associations with differential susceptibility to *Mycobacterium* infection [8], [12], [13], [14]. The role of TLR signaling in mycobacterial infection has been described in increased susceptibility of TLR2 and MyD88 knockout mice [15], [16], [17]. Recent studies have also indicated that specific TLR genes were differentially expressed in sheep infected or not with *M. avium* subsp. *paratuberculosis*, with TLR9 showing the highest difference [18], [19], [20], [21]. On the other hand and to the best of our knowledge, no polymorphisms in TLR genes potentially associated with *Small Ruminant Lentivirus* or *C. abortus* infection have been reported.

TLR9 is expressed in the endosomal compartments of dendritic cells and macrophages. The functional receptor is present as a preformed dimer, recognizes unmethylated CpG dinucleotides in bacterial and viral DNA through the ectodomain and undergoes proteolytic cleavage of its N-terminal in order to be activated. As suggested, cleavage takes place in the spacer region between LRR14 and LRR15 of the ectodomain, a site, which conforms a flexible loop, possibly exposed to proteolysis [22], [23]. Proteolysis of the TLR9 is necessary for subsequent signal transduction through the adaptor protein MyD88 [22], [23], in order to activate the NF-κB pathway.

The study described here was designed and performed based on previous findings regarding involvement of TLR9 in susceptibility of sheep to infections. Objectives of the study were to evaluate levels of nucleotide variation of *TLR9* and its mediator *MyD88* gene in three sheep flocks, derived from different breeds and to assess their potential association with seropositivity for *Small Ruminant Lentivirus*, *C. abortus* or *M. avium* subsp. *paratuberculosis*.

## Materials and Methods

### Animals

Samples from 115 sheep (*Ovis aries*) of the following three breeds: Boutsko-breed (n = 56), Friesarta-breed (n = 27) and Comisana-breed (n = 32 samples), were used in the study. Boutsko-breed is indigenous in Greece and includes animals with low production, usually farmed extensively in the mountains and arid areas of the country. Friesarta-breed is a cross breed, with high-yielding animals, farmed intensively in the lowlands. Comisana-breed is indigenous in Italy and includes animals with medium to high production, farmed in a variety of production systems. Samples were collected from three flocks, each one with animals of only one of the above breeds. All animals from which samples were collected, were older than 3 years.

All farms from animals of which samples were collected, were commercial flocks, managed semi-intensively; the flocks were under veterinary care, which was provided by private practicing veterinarians. No animals were euthanised during the study and efforts taken to ameliorate animal suffering. The study did not involve any experimentation, but was based in blood samples, that had been collected from the sheep for routine diagnostic purposes in the participating flocks. Diagnostic veterinary procedures are not within the context of relevant EU legislation for animal experimentations (Directive 86/609/EC) and may be performed in order to diagnose animal diseases and improve animal welfare.

### Detection of Seropositivity for Small Ruminant Lentivirus, C. abortus or ***M. avium*** subsp. ***paratubercul***osis

For the detection of seropositive animals for *Small Ruminant Lentivirus*, blood samples were collected and tested for *Small Ruminant Lentivirus* antibodies by using a commercially available ELISA kit (IDDEX). The initial findings were subsequently confirmed by the PCR technique, where EDTA-treated blood samples from seropositive sheep were tested. Proviral DNA was extracted from blood samples, using the Blood kit for RNA and DNA purification (Gentra Systems). Extractions were carried out according to the manufacturer’s instructions. The final yield of DNA products was stored at −20°C, until use. Primers for a nested PCR, specific for the *gag-pol* and the *pol* gene region [24], were used to amplify a 1.8 kb and a 1.2 kb sequence, respectively. PCR products were gel-purified (QIAquick Gel Extraction Kit; Qiagen Ltd) and sequence analysis was performed twice on the complete viral genome (MWG Biotech), by using the forward and reverse PCR primers. All samples were analysed twice and only high-quality sequences were used.

The CHECKIT™ Chlamydia Test Kit (IDDEX) was used for detection of *C. abortus* antibodies in blood samples. All procedures were carried out according to the manufacturer’s instructions. Measurements were performed in duplicate and matching serum pairs were analyzed on the same microtitre plate. Samples with values <30% were considered to originate from animals with no infection, samples with values between 30 and 40% were considered to be from animals with inconclusive infection status and samples with values >40% were considered to originate from infected animals.

*M. avium* subsp. *paratuberculosis* diagnosis was performed in blood serum samples by testing for antibodies to the organism with a commercial ELISA kit (IDEXX) using the manufacturer’s protocol. Additionally, faecal samples were collected and cultured, following a slight modification of the protocol described in [25]. Specifically, the cultures were incubated at 37°C for up to 30 weeks and examined every fortnight for bacterial growth. To confirm the identity of colonies isolated, DNA was extracted from the colonies and screened for presence of the *M. avium* subsp. *paratuberculosis*-specific insertion sequence IS*900* [26].

### Isolation of Sheep Genomic DNA and PCR Amplification

Total genomic DNA was extracted from blood samples in the presence of 20 mM EDTA and was preserved in −20°C, according to [27]. PCR reactions (50 µL) contained 200–300 ng of genomic DNA, 10× Taq buffer, 2 mM MgCl_2_, 0.2 mM of each dNTP, 25 pmoles of each primer and 1 U Taq polymerase (Invitrogen). The primers (Fig. S1) were designed based on the TLR9 and MyD88 full length cDNA sequences (Genbank ID: NM_001011555.1, [28]) and NM_001166183, respectively]. For TLR9, the PCR product (414 bp) corresponded to nucleotides 1344–1757 of NM_001011555.1 sequence (Fig. S1). MyD88 PCR product contained nucleotides 470–738 of NM_001166183 sequence and, additionally, one intron of 170 bp (Fig. Sl). PCR conditions included an initial denaturation at 95°C for 5 min, followed by 35 cycles of denaturation at 95°C for 30 sec, annealing at 59°C for 40 sec and extension at 72°C for 40 sec, with a final extension at 72°C for 10 min. SSCP analysis has been performed as previously described in [27]. Homozygous samples were directly sequenced bi-directionally by Macrogen Inc., while heterozygous samples were sequenced after cloning in pGEM-T Easy vector.

### Data Analysis

The nucleotide and amino acid sequences were analyzed using the Bioedit v. 7.0.4 software [29]. Expected (*H*_e_) and observed (*H*_o_) heterozygosity were estimated using the GENETIX software [30]. Frequency distributions of each allele and genotype in seropositive or seronegative animals for each of the three agents under study were compared pair-wise with the *χ* ^2^ test using SPSS, which was used for performing all necessary analyses. Significance was set as *P*<0.05.

## Results

### Variation in *TLR9* and *MyD88* Genes

For *TLR9*, the PCR product corresponded to part of LRR 14, the spacer region between LRR 14 and 15 and the entire LRR 15, 16, 17 and 18 [11]. It has been selected, because it included the *TLR9* cleavage site [23], wherein putative polymorphisms might be significant. In total, seven *TLR9* alleles were identified (Fig. S1), five of which (TLR9-05 to TLR9-09) were new (Genbank accession numbers: HQ717158, HQ717159, HQ717160, JN377802, JN377803). The remaining two alleles (TLR9-10 and TLR9-11) were identical to alleles previously identified in Romney-breed animals (EF656459.1, EF656458.1). The nucleotide substitutions resulted in two synonymous and three non-synonymous nucleotide substitutions, which changed the amino-acid polarity (Table 1, Fig. S1). More specifically, two of the amino acid changes (R447Q, A462S) were found to be located in the spacer region of the TLR9 protein and the third (G520R) in the first residue of the LRR17 (Table 1, Fig. S1).

**Table 1:** SNPs and alleles identified in ovine *TLR9* and *MyD88* genes.

SNP^a^	Alleles^b^									Amino-acid change	Charge change
	*05*	*06*	*07*	*08*	*09*	*10*	*11*	*01*	*02*		
C1323T	C	C	T	C	C	C	T			No change	No change
G1340A	G	A	G	A	G	G	G			R447Q	Positive/polar
G1384T	G	T	G	T	T	G	G			A462S	Non-polar/polar
G1557C	G	G	G	C	G	C	C			G520R	Non-polar/positive
T1571C	T	T	C	C	T	C	C			No change	No change
T528C								T	C	No change	No change
A570G								A	G	No change	No change

a Numbering corresponds to NM_001011555.1 *TLR9* and to NM_001166183.1 *MyD88* sequences, counting as position 1 the nucleotide A of the ATG codon.

b Alleles 05–11 correspond to *TLR9* and alleles 01–02 to *MyD88*.

For *MyD88*, the PCR product contained the coding region for the TIR domain, which is responsible for MyD88 sorting and signaling. *MyD88* gene exhibited less polymorphism than *TLR9*; only two different alleles (MyD88_01, MyD88_02) (Fig. S1) were identified (Genbank accession numbers: HQ717161, HQ717162) in 74 animals. The two nucleotide substitutions, which were identified, were synonymous and were localized in exonic sequences (Table 1, Fig. S1).

### Allelic Frequencies

Calculation of allelic frequencies in the three breeds studied, showed that *TLR9-10* allele was the most common, with a frequency range of 45%–68.5% (Table 2). However, frequencies of the remaining alleles showed significant differences between breeds; allele *TLR9-05* had a frequency of 21.5% and 40% in Boutsko and Comisana breeds, respectively, and was absent from Friesarta-breed (Table 2). For *MyD88* gene, allele *MyD88_02* was the most frequently identified in all breeds.

**Table 2:** Allele frequency of *TLR9* and *MyD88* genes in three sheep breeds.

Breed	Allele frequency (%) for *TLR9*							Allele frequency (%) for *MyD88*	
	*05*	*06*	*07*	*08*	*09*	*10*	*11*	*01*	*02*
Boutsko	22	8	2	2	0	49	18	20	80
Friesarta	0	19	0	0	0	69	13	4	96
Comisana	40	0	10	2	3	45	0	21	79

### Seroprevalence of SRLV, MAP and *Chlamydophila abortus*

Seroprevalence of *Small Ruminant Lentivirus*, *C*. *abortus* and *M. avium* subsp. *paratuberculosis* infection was 39%, 8% and 10%, respectively. However, seroprevalence of each of the above infective agents differed significantly between the three flocks. Details are in Table 3.

**Table 3:** Expected (*H_e_*) and observed (*H_o_*) heterozygosity for *TLR9* gene, in relation to seropositivity for *Small Ruminant Lentivirus* (*SRLV*), *C. abortus* (*CA*) or *M. avium* subsp. *paratuberculosis* (*MAP*) in three sheep flocks from different breeds.

Breed	*H_e_*	*H_o_*	Seroprevalence		
			*SRLV*	*CA*	*MAP*
Boutsko	0.675	0.843	41%	6%	8%
Friesarta	0.479	0.259	70%	11%	19%
Comisana	0.626	0.900	7%	10%	7%
Total			39%	8%	10%

### Levels of Heterozygosity for *TLR9* Alleles

Observed heterozygosity was found to be higher than expected in Boutsko and Comisana flocks; the opposite was identified in Friesarta flock (Table 3).

### Association of *TLR9* Genotypes with Seropositivity

The number of seropositive and seronegative animals for *Small Ruminant Lentivirus*, *C. abortus* or *M. avium* subsp. *paratuberculosis* in the three flocks according to *TLR9* genotypes are shown in Table 4 and in detail in Table S1. All sheep homozygote for TLR9_6 allele or heterozygote for TLR9_6/TLR9_5 or TLR9_6/TLR9_10 were found to be seronegative. Additionally, 96% of animals with the TLR9_5 allele together with the TLR9_10 or the TLR9_6 allele were also found seronegative. The TLR9_5 allele has not been found in homozygosity. On the other hand, the homozygosity of TLR9_10 allele does not seem to be associated with seronegativity, as only three out of nineteen individuals are seronegative.

**Table 4:** Association of *ΤLR9* genotypes with seroprevalence for *Small Ruminant Lentivirus* (*SRLV*), *C. abortus* (*CA*) or *M. avium* subsp. *paratuberculosis* (*MAP*) in three sheep flocks from different breeds.

Breed		Number of animals according to seropositive status					
	Genotype	*SRLV* +	*SRLV* −	*CA* +	*CA* −	*MAP* +	*MAP* −
Boutsko	TLR9_6	0	3	0	3	0	3
	TLR9_7	0	1	0	1	0	1
	TLR9_10	1	3	1	3	1	3
	TLR9_5+ TLR9_6	0	1	0	1	0	1
	TLR9_5+ TLR9_10	0	21	0	21	0	21
	TLR9_6+ TLR9_10	0	1	0	1	0	1
	TLR9_8+ TLR9_10	2	0	0	2	0	2
	TLR9_10+ TLR9_11	18	0	2	16	3	15
Friesarta	TLR9_6	0	5	0	5	0	5
	TLR9_10	12	3	0	15	3	12
	TLR9_10+ TLR9_11	7	0	2	5	3	4
Comisana	TLR9_7	0	3	2	1	0	3
	TLR9_5+ TLR9_10	0	24	0	24	1	23
	TLR9_8+ TLR9_10	1	0	0	1	0	1
	TLR9_9+ TLR9_10	1	1	0	2	1	1
Cumulative results	TLR9_6	0	8	0	8	0	8
	TLR9_7	0	4	2	2	0	4
	TLR9_10	13	6	1	18	4	15
	TLR9_5+ TLR9_6	0	1	0	1	0	1
	TLR9_5+ TLR9_10	0	45	0	45	1	44
	TLR9_6+ TLR9_10	0	1	0	1	0	1
	TLR9_8+ TLR9_10	3	0	0	3	0	3
	TLR9_9+ TLR9_10	1	0	0	1	0	1
	TLR9_10+ TLR9_11	25	0	4	21	6	19

+: seropositive animals,

−: seronegative animals.

Comparison of the non-synonymous substitutions between the various alleles (Table 1) showed that the G520R substitution was the one differentiating them; at amino-acid level, the allele TLR9_5 was identical to TLR9_7 and TLR9_10 was identical to TLR9_11. In addition, both alleles TLR9_5 and TLR9_10 carry arginine (R) and alanine at positions 447 and 462, respectively. The allele and genotype frequencies of the G520R in seropositive and seronegative animals are shown in Table 5. Frequency of *R* variation was significantly increased in sheep seropositive for *Small Ruminant Lentivirus* (*P*<0.001). Frequency of *RR* genotypes was also significantly increased in seropositive sheep, as identified based on recessive (*P*  = 0.004) or genotypic (*P*  = 0.009) model. Finally, no associations were identified for allele or genotype frequencies between *C. abortus*- or *M. avium* subsp. *paratuberculosis*-seropositive or seronegative animals.

**Table 5:** Genotype frequency of the G520R substitution in sheeps seropositive or seronegative for *Small Ruminant Lentivirus* (*SRLV*), *C. abortus* (*CA*) or *M. avium* subsp. *paratuberculosis* (*MAP*).

Seropositive status	Genotype for G520 R				Allele frequency			Genotype frequency						
	*GG* or *GR*	*RR*	*GG*	*GR*	P(R)	P(G)	*P*	*GG* or *GR*	*RR*	*P*	*RR*	*GG*	*GR*	*P*
*SRLV* +	0.024	0.976	0	0.024	0.988	0.001	0.024	0.976			0.976	0	0.024	
*SRLV* **−**	0.909	0.091	0.197	0.712	0.447	0.553	<0.001	0.909	0.091	0.004	0.091	0.197	0.712	0.009
*CA* +	0.182	0.818	0.182	0	0.82	0.18	0.182	0.818			0.818	0.182	0	
*CA* **−**	0.608	0.392	0.113	0.495	0.64	0.36	0.114	0.608	0.392	0.433	0.392	0.113	0.495	0.607
*MAP* +	0.333	0.667	0	0.333	0.83	0.17	0.333	0.667			0.667	0.667	0.333	
*MAP* **−**	0.586	0.414	0.131	0.455	0.64	0.36	0.095	0.586	0.414	0.625	0.414	0.414	0.455	0.853

+: seropositive animals,

−: seronegative animals.

## Discussion

Until today, *TLR9* polymorphisms have been described only in two breeds, the Tsigai-breed, which is distributed in Eastern and Central Europe, and in Romney-breed, which is widely farmed in New Zealand [31], [32]. In the present study, seven different alleles were identified for *TLR9*, six of which were found to be present in the flock from Boutsko-breed, three in the flock from Friesarta-breed and five in the flock from Comisana-breed. Observed differences in the level of polymorphism and the absence of specific alleles from a breed may be the consequence of evolution forces, although sampling bias may also contribute as the sample selection does not allow for a good representation of the sheep population.

To the best of our knowledge, this is the first report regarding level of polymorphism of *MyD88* gene in sheep (*Ovis aries*). The gene exhibits substantially lower polymorphism; only two alleles, with no difference at the level of amino-acid sequence, were detected among the animals studied. This may be the consequence of the TIR domain containing three highly conserved motifs, which are essential for proper expression and signaling [33]. Nevertheless, during this study, one intron of 170 bp that follows the *GT*-*AG* rule was identified, according to the genomic organization of this gene in other mammals (e.g. Gene ID 444881 in *Bos taurus* and 17874 in *Mus musculus*).

A significant difference was found in seroprevalence among flocks; Friesarta-breed animals have a higher seroprevalence, especially for *Small Ruminant Lentivirus*, the seroprevalence of which has been found to be 70%, whilst in Boutsko- and Comisana-breeds it was 41% and 7%, respectively. Under the heterozygote advantage hypothesis, this could be attributed to the low levels of TLR9 observed heterozygosity in the flock from Friesarta-breed, where observed heterozygosity was significantly lower to the expected. This might be the effect of population bottlenecks, inbreeding or meta-population dynamics, which have reduced the level of genetic variation. Alternatively, the high seroprevalence might be the consequence of the presence of the G520R R allele in this breed (only 19% of the animals carried the G allele). The results also indicate that the TLR9_5 and TLR9_6 alleles are associated with seronegativity. Finally, we cannot exclude that the findings are a product of chance due to the sampling bias. It is noteworthy that animals of Friesarta-breed have been reported to have an increased susceptibility to bacterial respiratory infections [34]. This could be the result of subclinical *Small Ruminant Lentivirus* infections, which have a predisposing role to bacterial infections of the lungs. Moreover, Friesarta-breed sheep have been previously reported to have increased susceptibility to bacterial mastitis [35].

Moreover, our results indicated that the change of G to R at codon 520 could be associated with seropositivity for *Small Ruminant Lentivirus*. Potential associations between various polymorphisms of TLR9 and *Small Ruminant Lentivirus* susceptibility have been previously studied [32], but the results obtained were not conclusive enough regarding such an association. This amino-acid substitution, which can result in polarity change, might influence structure and function of LRR17, interfering with ligand binding, and could be used in studies examining susceptibility/resistance to *Small Ruminant Lentivirus* infections in sheep. Further work is needed in which more flocks from different breeds will be analyzed, in order to have a better representation of the sheep population. In addition, functional studies are required in order to identify whether this variant itself affects TLR9 function or it is an indirect marker of association due to linkage disequilibrium.

## Supporting Information

Figure S1 ***TLR9*** **and** ***MyD88*** **polymorphisms in** ***Ovis aries*** **.** DNA and protein sequence alignment for the seven *TLR9* and the two *MyD88* alleles that were identified in this study (Genbank accession numbers: HQ717158, HQ717159, HQ717160, JN377802, JN377803 and HQ717161, HQ717162, respectively). Dots indicate bases that are identical at the position to top sequence. Primer sequences are shown underlined. The limits of the different LRRs (part of LRR14, LRR15-LRR17 and part of LRR18) were defined according to human orthologous protein (Bell *et al.* 2003) and are shown by arrows. The intervening region between LRR14 and 15 is shaded in gray.
(DOC)

Table S1 **Genotypes in** ***TLR9*** **gene in sheep breeds.**
(XLSX)
