# Supplementary material for: Toll Like Receptor 9 (TLR9) Polymorphism G520R in Sheep Is Associated with Seropositivity for Small Ruminant Lentivirus
Source: PLoS One. 2013 May 15;8(5):e63901. doi: 10.1371/journal.pone.0063901 (PMC3655008; doi:10.1371/journal.pone.0063901)
Supplement: Figure S1 — TLR9 and MyD88 polymorphisms in Ovis aries . DNA and protein sequence alignment for the seven TLR9 and the two MyD88 alleles that were identified in this study (Genbank accession numbers: HQ717158, HQ717159, HQ717160, JN377802, JN377803 and HQ717161, HQ717162, respectively). Dots indicate bases that are identical at the position to top sequence. Primer sequences are shown underlined. The limits of the different LRRs (part of LRR14, LRR15-LRR17 and part of LRR18) were defined according to human orthologous protein (Bell et al. 2003) and are shown by arrows. The intervening region between LRR14 and 15 is shaded in gray. (DOC) [file pone.0063901.s001.doc]

***TLR9-05*** 1 **ttc gtg gac ctg tcg gac aac cgc atc agc gga gct gcg agg ccg gtg gcc gcc ctc ggg gag gtg gac agc ggg gtg gaa gtc tgg cgg tgg ccc agg ggc ctc gct cca**

**F V D L S D N R I S G A A R P V A A L G E V D S G V E V W R W P R G L A P**

***TLR9-06*** 1 **... ... ... ... ... ... ... ... ... ... ... ... ... ... ... ... ... ... ... ... ... ... ... ... ... ... ... ... ... .a. ... ... ... ... ... ... ...**

. . . . . . . . . . . . . . . . . . . . . . . . . . . . . **Q** . . . . . . .

***TLR9-07*** 1 **... ... ... ... ... ... ... ... ... ... ... ... ... ... ... ... ... ... ... ... ... ... ... ..t ... ... ... ... ... ... ... ... ... ... ... ... ...**

. . . . . . . . . . . . . . . . . . . . . . . . . . . . . . . . . . . . .

***TLR9-08*** 1 **... ... ... ... ... ... ... ... ... ... ... ... ... ... ... ... ... ... ... ... ... ... ... ... ... ... ... ... ... .a. ... ... ... ... ... ... ...**

. . . . . . . . . . . . . . . . . . . . . . . . . . . . . **Q** . . . . . . .

***TLR9-09*** 1 **... ... ... ... ... ... ... ... ... ... ... ... ... ... ... ... ... ... ... ... ... ... ... ... ... ... ... ... ... ... ... ... ... ... ... ... ...**

. . . . . . . . . . . . . . . . . . . . . . . . . . . . . . . . . . . . .

***TLR9-10*** 1 **... ... ... ... ... ... ... ... ... ... ... ... ... ... ... ... ... ... ... ... ... ... ... ... ... ... ... ... ... ... ... ... ... ... ... ... ...**

. . . . . . . . . . . . . . . . . . . . . . . . . . . . . . . . . . . . .

***TLR9-11*** 1 **... ... ... ... ... ... ... ... ... ... ... ... ... ... ... ... ... ... ... ... ... ... ... ..t ... ... ... ... ... ... ... ... ... ... ... ... ...**

. . . . . . . . . . . . . . . . . . . . . . . . . . . . . . . . . . . . .

***TLR9-05*** 112 **ggc ccg ctg gcc gcc gtc agc gca aag gac ttc atg cca agc tgc aac ctc aac ttc acc ttg gac ctg tca cgg aac aac ctg gtg acg atc cag cag gag atg ttt acc**

**G P L A A V S A K D F M P S C N L N F T L D L S R N N L V T I Q Q E M F T**

***TLR9-06*** 112 **... ... ... ... ... ... ... t.. ... ... ... ... ... ... ... ... ... ... ... ... ... ... ... ... ... ... ... ... ... ... ... ... ... ... ... ... ...**

. . . . . . . **S** . . . . . . . . . . . . . . . . . . . . . . . . . . . . .

***TLR9-07*** 112 **... ... ... ... ... ... ... ... ... ... ... ... ... ... ... ... ... ... ... ... ... ... ... ... ... ... ... ... ... ... ... ... ... ... ... ... ...**

. . . . . . . . . . . . . . . . . . . . . . . . . . . . . . . . . . . . .

***TLR9-08*** 112 **... ... ... ... ... ... ... t.. ... ... ... ... ... ... ... ... ... ... ... ... ... ... ... ... ... ... ... ... ... ... ... ... ... ... ... ... ...**

. . . . . . . **S** . . . . . . . . . . . . . . . . . . . . . . . . . . . . .

***TLR9-09*** 112 **... ... ... ... ... ... ... t.. ... ... ... ... ... ... ... ... ... ... ... ... ... ... ... ... ... ... ... ... ... ... ... ... ... ... ... ... ...**

. . . . . . . **S** . . . . . . . . . . . . . . . . . . . . . . . . . . . . .

***TLR9-10*** 112 **... ... ... ... ... ... ... ... ... ... ... ... ... ... ... ... ... ... ... ... ... ... ... ... ... ... ... ... ... ... ... ... ... ... ... ... ...**

. . . . . . . . . . . . . . . . . . . . . . . . . . . . . . . . . . . . .

***TLR9-11*** 112 **... ... ... ... ... ... ... ... ... ... ... ... ... ... ... ... ... ... ... ... ... ... ... ... ... ... ... ... ... ... ... ... ... ... ... ... ...**

***TLR9-05*** 223 **cgc ctc tcc cgc ctc cag tgc ctg cgc ctg agc cac aac agc atc tcg cag gcg gtt aat ggc tcg cag ttc gtg ccg ctg acc ggc ctg cga gtg ctt gac ctg tcc** **tac**

**R L S R L Q C L R L S H N S I S Q A V N G S Q F V P L T G L R V L D L S Y**

***TLR9-06*** 223 **... ... ... ... ... ... ... ... ... ... ... ... ... ... ... ... ... ... ... ... ... ... ... ... ... ... ... ... ... ... ... ... ... ... ... ...** ...

. . . . . . . . . . . . . . . . . . . . . . . . . . . . . . . . . . . . .

***TLR9-07*** 223 **... ... ... ... ... ... ... ... ... ... ... ... ... ... ... ... ... ... ... ... ... ... ... ... ... ... ... ... ... ... ... ... ..c ... ... ...** ...

. . . . . . . . . . . . . . . . . . . . . . . . . . . . . . . . . . . . .

***TLR9-08*** 223 **... ... ... ... ... ... ... ... ... ... ... ... ... ... ... ... ... ... ... ... ... ... ... ... ... ... ... ... c.. ... ... ... ..c ... ... ...** ...

. . . . . . . . . . . . . . . . . . . . . . . . . . . . **R** . . . . . . . .

***TLR9-09*** 223 **... ... ... ... ... ... ... ... ... ... ... ... ... ... ... ... ... ... ... ... ... ... ... ... ... ... ... ... ... ... ... ... ... ... ... ...** ...

. . . . . . . . . . . . . . . . . . . . . . . . . . . . . . . . . . . . .

***TLR9-10*** 223 **... ... ... ... ... ... ... ... ... ... ... ... ... ... ... ... ... ... ... ... ... ... ... ... ... ... ... ... c.. ... ... ... ..c ... ... ...** ...

. . . . . . . . . . . . . . . . . . . . . . . . . . . . **R** . . . . . . . .

***TLR9-11*** 223 **... ... ... ... ... ... ... ... ... ... ... ... ... ... ... ... ... ... ... ... ... ... ... ... ... ... ... ... c.. ... ... ... ..c ... ... ...** ...

***TLR9-05*** 334 **aac aag ctg gac ctg tac cat ggg cgc tcg ttc acg gag ctg ccg cag ctg gag gca ctg gac ctc agc tac aac agc cag**

**N K L D L Y H G R S F T E L P Q L E A L D L S Y N S Q**

***TLR9-06*** 334 **... ... ... ... ... ... ... ... ... ... ... ... ... ... ... ... ... ... ... ... ... ... ... ... ... ... ...**

. . . . . . . . . . . . . . . . . . . . . . . . . . .

***TLR9-07*** 334 **... ... ... ... ... ... ... ... ... ... ... ... ... ... ... ... ... ... ... ... ... ... ... ... ... ... ...**

. . . . . . . . . . . . . . . . . . . . . . . . . . .

***TLR9-08*** 334 **... ... ... ... ... ... ... ... ... ... ... ... ... ... ... ... ... ... ... ... ... ... ... ... ... ... ...**

. . . . . . . . . . . . . . . . . . . . . . . . . . .

***TLR9-09*** 334 **... ... ... ... ... ... ... ... ... ... ... ... ... ... ... ... ... ... ... ... ... ... ... ... ... ... ...**

. . . . . . . . . . . . . . . . . . . . . . . . . . .

***TLR9-10*** 334 **... ... ... ... ... ... ... ... ... ... ... ... ... ... ... ... ... ... ... ... ... ... ... ... ... ... ...**

. . . . . . . . . . . . . . . . . . . . . . . . . . .

***TLR9-11*** 334 **... ... ... ... ... ... ... ... ... ... ... ... ... ... ... ... ... ... ... ... ... ... ... ... ... ... ...**

MyD88-01 1 **TAG CCT GAG TAT TTT GAT GCC TTC ATC TGC TAC TGC CCC AGC GAT ATT GAG TTT GTC CAT GAG ATG ATC CGG CAG CTG GAA CAG ACA AAC TAT CGG CTG AAA TTG TGC GTG**

MyD88-02 1 **... ... ... ... ... ... ... ... ... ... ... ... ... ... ... ... ... ... ... ..C ... ... ... ... ... ... ... ... ... ... ... ... ... ..G ... ... ...**

MyD88-01 112 **TCT GAC CGT GAC GTC CTG CCT GGC ACC TGT GTC TGG TCC ATC GCC AGT GAA CTC ATT GAG AAG AGg ttg gct atg tgg cca cag ggc aag tgg gtg ggt gtg caa agc cct**

MyD88-02 112 **... ... ... ... ... ... ... ... ... ... ... ... ... ... ... ... ... ... ... ... ... ... ... ... ... ... ... ... ... ... ... ... ... ... ... ... ...**

MyD88-01 221 **gcc agg gat ccc cat gct ggg gcc ctc cca gtc agc ccc tgt cta gcc tgg cat act ggg atc ctc cca agg ctg tcc cct ggt gag tca cca cag tgc ctg tag cct gcc**

MyD88-02 221 **... ... ... ... ... ... ... ... ... ... ... ... ... ... ... ... ... ... ... ... ... ... ... ... ... ... ... ... ... ... ... ... ... ... ... ... ...**

MyD88-01 331 **cac tct ccc cta gGT GCC GTC GGA TGG TGG TGG TTG TCT CTG ACG AAT ACC TGC AAA GCA AGG AAT GTG ACT TCC AGA CTA AGT TTG CGC TCA GCC TCT CTC CAG GTA**

MyD88-02 331 **... ... ... ... ... ... ... ... ... ... ... ... ... ... ... ... ... ... ... ... ... ... ... ... ... ... ... ... ... ... ... ... ... ... ... ...**
